# Aerobic exercise decreases chemerin/CMKLR1 in the serum and peripheral metabolic organs of obesity and diabetes rats by increasing PPARγ

**DOI:** 10.1186/s12986-019-0344-9

**Published:** 2019-03-05

**Authors:** Xiaojing Lin, Yanan Yang, Jing Qu, Xiaohui Wang

**Affiliations:** School of Kinesiology, Shanghai University of Sport, 188 Hengren Road, Yangpu District, Shanghai, 200438 China

**Keywords:** Aerobic exercise, Chemerin, CMKLR1, PPARγ, Type 2 diabetes, Obesity

## Abstract

**Objective:**

To investigate the influences of exercise on the levels of chemerin and its receptor chemokine-like receptor (CMKLR1) in the peripheral metabolic organs of obesity and diabetes rats, and whether the mechanism is related to peroxisome proliferator activated receptor γ (PPARγ), a key modulator of glycolipid metabolism.

**Methods:**

Obesity rats induced by 8-week high fat diet (HFD) were randomly divided into obesity group (OB) and exercised obesity group (EOB) with 8 rats each group, and 40 diabetes rats established by 8-week HFD plus low dose of streptozotocin were randomly divided into 4 groups: diabetes group (DM), exercised diabetes group (EDM), exercised diabetes plus PPARγ agonist pioglitazone group (EDP), and exercised diabetes plus PPARγ antagonist GW9662 group (EDG). The rats in EOB, EDM, EDG and EDP groups participated in a 4-week moderate-intensity aerobic exercise on a treadmill with gradually increasing intensity, once a day and 6 days/week, and 30 min before each exercise EDP and EDG were administrated to the rats in EDP and EDG groups, respectively. Before and after 4-week exercise, glycolipid metabolism indexes, serum chemerin and the levels of chemerin and CMKLR1 in metabolic organs such as liver and gastrocnemius were investigated (not detecting adipose for no available perirenal adipose from DM rats).

**Results:**

(1) In addition to serum chemerin, the levels of chemerin and CMKLR1 in the liver and gastrocnemius of EOB and EDM rats were declined, accompanied with the improved glycolipid metabolism. (2) The decreased chemerin/CMKLR1 in the EDM rats were reversed by PPARγ antagonist GW9662 and further strengthened by PPARγ agonist pioglitazones.

**Conclusions:**

Besides serum chemerin, the levels of chemerin/CMKLR1 in the metabolic organs of obesity and diabetes rats were alleviated by exercise, which were likely to be associated with the improvement of glycolipid metabolism. Exercise-induced decrements of chemerin/CMKLR1 in the diabetes rats were mediated by PPARγ.

**Electronic supplementary material:**

The online version of this article (10.1186/s12986-019-0344-9) contains supplementary material, which is available to authorized users.

## Introduction

Obesity and its related diseases such as type 2 diabetes, metabolic syndrome and cardiovascular disease have been considered as a chronic low-grade systemic inflammation that is associated with abnormal concentrations of inflammatory cytokines and adipokines [1]. In addition to classic inflammatory cytokines and adipokines, chemerin, a novel adipokine has attracted more attentions. Chemerin is primarily produced in liver and adipose tissue and exerts important roles in multiple aspects such as adipogenesis, lipolysis, glycolipid metabolism, insulin resistance (IR), inflammation [2], energy metabolism, immunity, and cell proliferation and differentiation [3]. Circulating chemerin is elevated in numerous metabolic and inflammatory diseases including obesity [4], metabolic syndrome [5], type 2 diabetes [6], atherosclerosis [7, 8], hypertension [9], cardiovascular disease [8, 10] and nonalcoholic fatty liver disease [11, 12], and the chemerin level is associated with symptoms severity of these inflammatory diseases [13].

Most functions of chemerin are mediated by its receptor, chemokine-like receptor (CMKLR1 or ChemR23) [14, 15], and CMKLR1 becomes a potential target in management of chemerin-induced IR and diabetes [16]. CMKLR1(−/−) mouse has lower food consumption, total body mass and percent body fat, and also exhibits decreased inflammation in hepatic and white adipose tissue compared with wild-type controls, regardless of low or high fat diet [14]. Thus, chemerin/CMKLR1 may be promising new targets for the treatment of obesity and its related diseases, and peptides or other substances that affect chemerin/CMKLR1 axis will be used in the future in the treatment of obesity and diabetes [15].

Exercise has positive effects on reducing obesity, and prevention and treatment of obesity related diseases [17, 18]. The benefits of exercise on improvement of glycolipid metabolism and suppression of inflammation have been confirmed in obese adults [19] and adolescents [20], but the underlying mechanisms are not fully clarified. A significant exercise-induced decline in circulating chemerin has been demonstrated by many reported [21–23], and the decrease of circulating chemerin is likely to be connected with the improvement of glycolipid metabolism and the decrease of inflammation, not only in normal elderly [24] but also in obese [25, 26] and diabetic patients [27]. However, whether the levels of chemerin and CMKLR1 in peripheral metabolic organs are altered by exercise in obesity and diabetes are still unknown, not to speak of the underlying mechanism.

Peroxisome proliferator-activated receptor (PPAR)γ, a ligand-activated nuclear receptor, has been identified as a therapeutic target for obesity, hyperlipidemia and diabetes for the dual functions of regulating glycolipid metabolism [28, 29] and inhibiting inflammation [30, 31]. Exercise increases PPARγ in circulating monocytes of human [31] and skeletal muscle of diabetic Zucker rats [32], which may be related to the improvement of IR and glucose uptake of skeletal muscle. It’s worth mentioning that there is a putative PPARγ response element (PPRE) within chemerin promoter and a direct association of PPARγ with this PPRE, and chemerin is considered as a novel PPARγ target gene in promoting mesenchymal stem cell adipogenesis in vitro [33]. However, whether the regulation of PPARγ on chemern exists in vivo, especially in exercise status, has not been reported.

Therefore, the purpose of the current study was to verify: 1) if exercise reduced chemerin and CMKLR1in the peripheral metabolic organs (not only serum chemerin) of obesity and diabetes rats; 2) if exercise-induced decreases of chemerin/CMKLR1 were mediated by PPARγ in the diabetes rats.

## Material and methods

### Animals

One hundred and two male Sprague-Dawley (SD) rats (190–210 g weight) were purchased from Beijing Vital River laboratory animal technology Co. Ltd., and housed under standard specific pathogen free (SPF) conditions with 12 h:12 h light and dark cycles and food and water were provided ad libitum.

### Establishment of obesity and type 2 diabetes rats and grouping

After acclimating to laboratory conditions for 3 days, the 102 rats were randomly divided into control group (Con, *n* = 8) and high fat diet (HFD) group (*n* = 94), which were fed ad libitum by 8-week standard diet (overall calories of 3.5 kcal/g) and HFD (mixing 2.5 g cholesterol, 1 g sodium cholate hydrate, 20 g sugar, 10 g fat and 66.5 g standard diet with overall calories of 4.5 kcal/g), respectively. After 8 weeks, the rats whose body weight exceeding 20% of the average weight of control rats were considered as obesity rats. Mimicking the whole features of type 2 diabetes in humans, the 56 rats from HFD group (excluding 22 obesity-resistance rats whose body weight lower than the average weight of control rats) were injected intraperitoneally with small dosage streptozotocin (STZ) (Sigma, St. Louis, MO, USA) at 30 mg/kg body weight. After an additional week (the 9th week from the starting of the experiment), the rats showed fasting hyperglycemia (fasting blood glucose, FBG > 11.1 mmol/L) at 3 and 7 day post-injection were involved in the study as diabetes rats.

Sixteen obesity rats were selected and randomly divided into obesity group (OB, *n* = 8) and exercised obesity group (EOB, n = 8). Forty diabetes mellitus rats (DM model) were successfully established and randomly divided into 4 groups of 10 rats each: diabetes mellitus group (DM), exercised diabetes mellitus group (EDM), EDM plus PPARγ agonist pioglitazone group (EDP), and EDM plus PPARγ antagonist GW9662 group (EDG).

### Exercise and administration of PPARγ agonist and antagonist

**Fig. 1:**
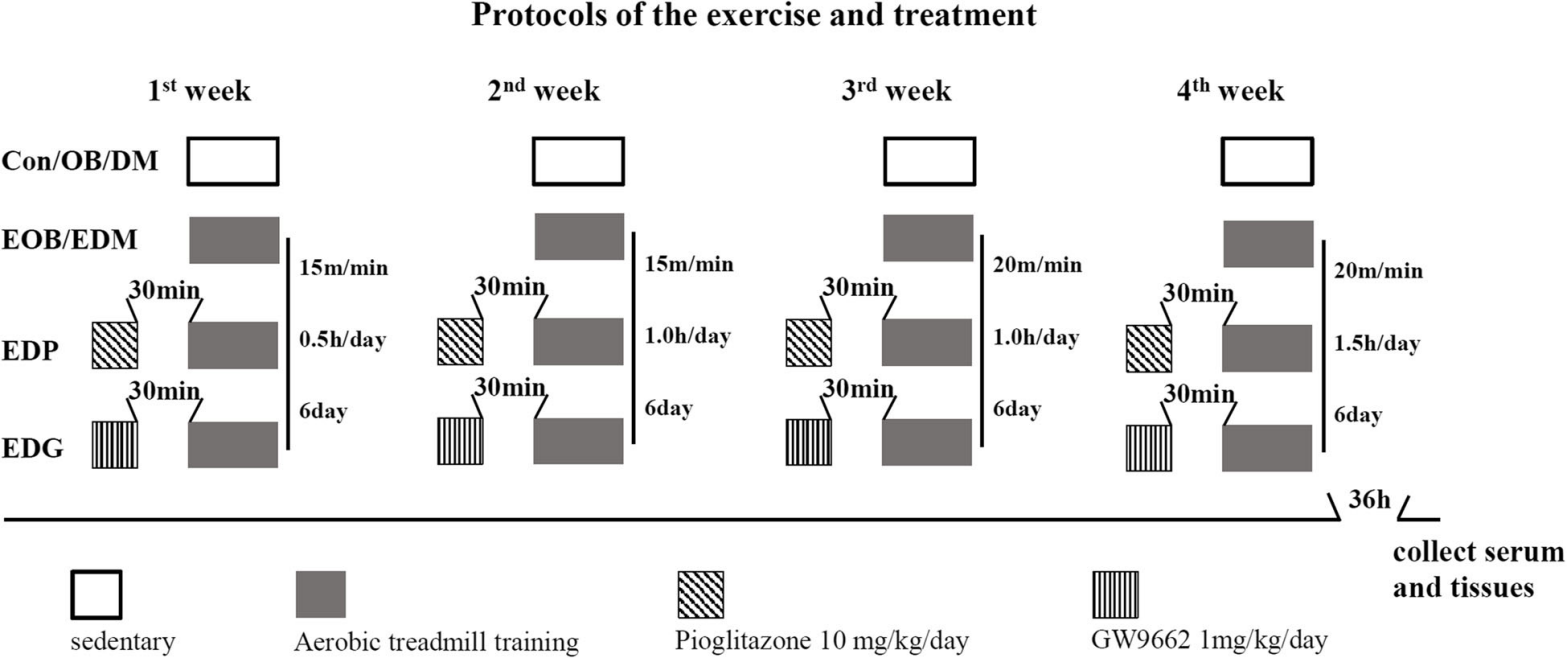
Protocols of the 4-week exercise and treatment with PPARγ agonist and antagonist. The rats in Con, OB and DM groups kept sedentary life while EOB, EDM, EDG and EDP rats participated in a moderate-intensity aerobic exercise on a treadmill with gradually increasing intensity and duration, once a day and 6 days/week, and at 30 min before exercise the rats in EDP and EDG groups were intragastrically administered PPARγ agonist pioglitazone (10 mg/kg body weight) and PPARγ antagonist GW9662 (1 mg/kg body weight) respectively. Con: control; OB: obesity; DM: diabetes mellitus; EOB: exercised OB; EDM: exercised DM. EDP: EDM + pioglitazone; EDG: EDM + GW9662

During the 4-week of exercise intervention, all the rats were fed with high fat diet. The rats in the groups of Con, OB and DM kept sedentary life while EOB, EDM, EDG and EDP rats participated in moderate-intensity aerobic exercise on a treadmill with gradually increasing intensity and duration, once a day and 6 days/week, and at 30 min before exercise the rats in EDP and EDG groups were intragastrically administered 10 mg/kg body weight of PPARγ agonist pioglitazone (MedChem Express, NJ, USA) and 1 mg/kg body weight of PPARγ antagonist GW9662 (MedChem Express, NJ, USA), respectively (Fig. 1). During the 4-week intervention period, one rat in EDG group was accidently squeezed to death in the gap between the runways of treadmill and six rats from the four groups died probably to be associated with diabetes, and finally 9, 8, 9 and 7 rats were involved in the analysis of results in DM, EDM, EDP and EDG groups, respectively.

### Determination of glycolipid metabolism index

Lipid metabolism index including serum triglyceride (TG), total cholesterol (TC), high-density lipoprotein cholesterol (HDL) and low-density lipoprotein cholesterol (LDL) were detected by Nanjing Jiancheng Bioengineering Institute. Glucose metabolism index such as FBG and fasting insulin (FINS) were measured by glucose meter (Roche Accu-Chek Performa, Germany) and ELISA, respectively. The variance coefficient of the rat insulin ELISA kit (Sigma-Aldrich, Germany) was <10% in intra-assay and <12% in inter-assay. IR was estimated by homeostasis model assessment of IR (HOMA-IR), calculating by the formula: = FBG (mmol/L) × FINS (μU/mL) / 22.5.

### Detection of serum chemerin

Serum levels of chemerin in the rats were measured by ELISA according to the manufacturer’s instruction. The variance coefficient of the rat chemerin ELISA Kit (LifeSpan BioSciences, Inc. WA, USA) was <10% in intra-assay and <12% in inter-assay.

### Quantitative real-time PCR

The rats were anaesthetized at 36 h after the last exercise, and liver and gastrocnemius of the rats were collected and frozen in liquid nitrogen until analyzed or immediately processed as described below. Total RNA was extracted using Trizol reagent (Invitrogen, CA, USA), and 4 μg of total RNA was subjected to synthesize first strand cDNA by Revert Aid First Stand cDNA Synthesis Kit (Thermo Scientific, MA, USA) in accordance with the manufacturer’s instructions. Primers for amplification genes of chemerin (sense: 5′-CGA GTG TCG GGA TTT AGT-3′, antisense: 5′-GGT AGG CAT CGT AGG TGA-3′), CMKLR1 (sense: 5′-CTA CCA CTG GGT GTT CGG-3′, antisense: 5′- GGG AGG AGC ACG GAG AT-3′), PPARγ (sense: 5′-ATC AGG TTT GGG CGA ATG-3′, antisense: 5′-TTT GGT CAG CGG GAA GG-3′), and GAPDH (sense: 5′-GCT GAG TAT GTC GTG GAG-3′, antisense: 5′-TCT TCT GAG TGG CAG TGA T-3′) were synthesized by Sangon Biotech Co, Ltd. shanghai. 50 ng of cDNA templates were added into FastStart universal SYBR Green Master (Roche company, Switzerland) to amplify the above genes, and the amplification processes of these genes were same: 10 min denaturation at 95 °C followed by 42 cycles of 15 s denaturation at 95 °C, 20 s annealing at 60 °C, 15 s elongation at 72 °C, and a final extension for 10 min at 72 °C. The mRNA values of the above target genes were calculated by the respective genes’ standard curve and corrected by the internal control of GAPDH, then presented as the ratio of the genes to GAPDH.

### Western blot

About 50 mg of liver and gastrocnemius were cut into pieces and homogenized with a homogenizer after adding 500 μL of radioimmunoprecipitation assay (RIPA) buffer containing 1 mmol/L phenylmethylsulfonyl fluoride (PMSF) (Beyotime Biotechnology, China) to extract total protein. The lysates were briefly sonicated on ice and centrifuged at 11963 g for 10 min. Supernatants were collected and protein concentration was measured using a BCA protein assay kit (Beyotime Biotechnology, Shanghai, China) according to the manufacturer’s instruction. Extracts (40 μg) of liver and gastrocnemius were fractionated on 10% SDS-PAGE gels for detecting all the molecules except chemerin (15% SDS-PAGE gel). The resolved proteins were electrotransfered onto nitrocellulose membranes, blocked with 5% nonfat milk for 2 h, and then incubated overnight at 4 °C with primary antibodies against chemerin (1:500), CMKLR1 and PPARγ (1:1000, abcam Company, UK), and β-actin and GAPDH (1:1000, Cell Signaling Technology, MA, USA). The blots were washed three times, for 5 min each time, with Tris-buffered saline with 0.1% Tween 20 (TBST) and incubated with horseradish peroxidase (HRP) conjugated secondary antibodies (1:5000, Santa Cruz Biotechnology, CA, USA) for 1 h at room temperature. The blots were washed again as described above, developed with Immobilon Western chemiluminescent HRP substrate (Millipore, MA, USA), and visualized by automatic chemiluminescence image analysis system (Tanon Biotechnology, Shanghai, China). The density of bands was determined using Bio-image software (Tanon Biotechnology, Shanghai, China) and normalized against β-actin or GAPDH.

### Statistical analysis

Statistical analysis of data was performed using SPSS for Windows 21.0 software package (IBM Corporation, Armonk, NY, USA). All data were expressed as mean ± SD, and the level of statistical significance was set as *p <* 0.05. Mean values in different groups with pre- and post- measures such as body weights, TC, TG, LDL, HDL, FBG, FINS and HOMA-IR were compared using repeated measures analysis of variance (ANOVA). Other data were analyzed using one-way ANOVA and post hoc comparisons using least significant difference (LSD)-t test.

## Results

### Successful establishment of obesity and diabetes model rats

**Fig. 2:**
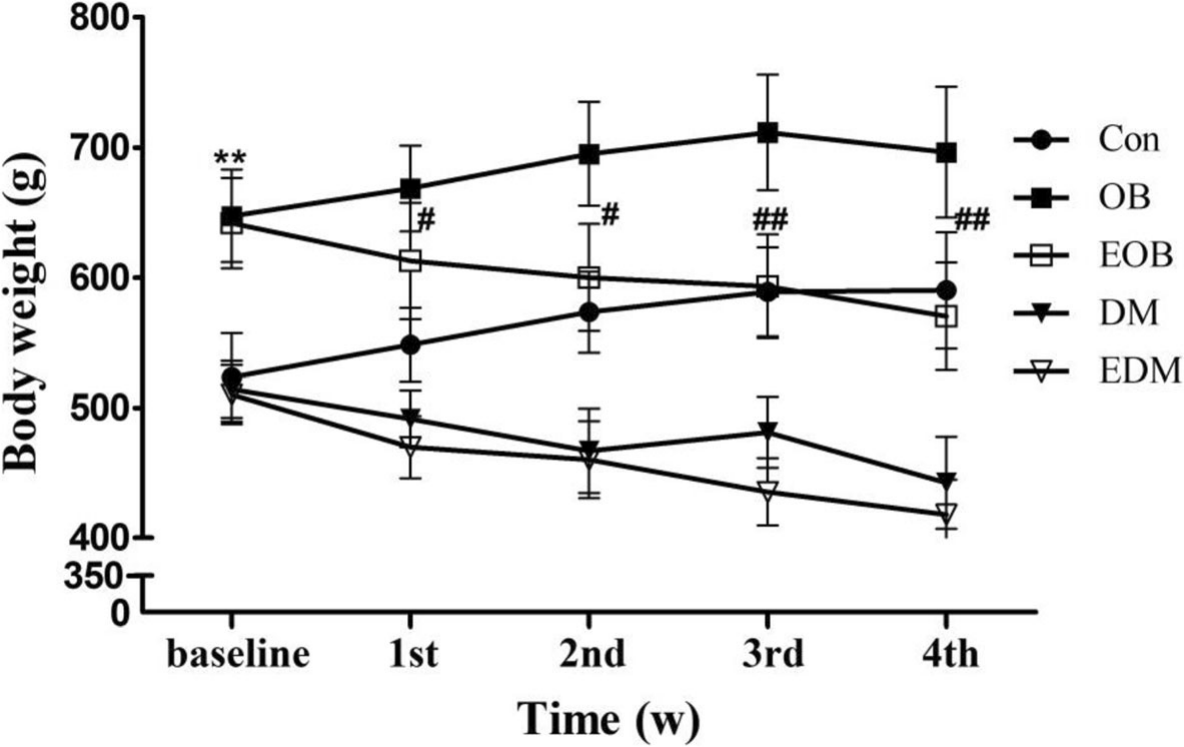
Influence of 4-week exercise on the body weights of obesity and diabetes rats. The body weights of Con, OB, EOB, DM and EDM rats were detected during 4-week exercise intervention. Con: control; OB: obesity; EOB: exercised OB; DM: diabetes mellitus; EDM: exercised DM. ^**^P<0.01 compared to Con at baseline condition; ^#^*P<*0.05; ^##^*P<*0.01 EOB vs OB

As shown in Fig.2, the mean body weights of OB model rats were increased by 1.24 fold (from 523.5 ± 33.9 g to 647.6 ± 35.7 g) exceeding the obesity criteria of 1.2 fold of the average weight of control rats, indicated the successful establishment of obesity model rats. For DM model rats, the mean FBG were enhanced by 16.4 mmol/L above the criteria of diabetes (FBG > 11.1 mmol/L). Furthermore, the DM rats had the symptoms of diabetes such as eating too much (daily intake: DM 67.0 ± 2.7 g vs Con 31.2 ± 2.6 g) and losing weight progressively (from baseline 514.3 ± 21.7 g to 4th week 442.4 ± 35.6 g) while the Con rats maintained the increase of weight. These results indicated the successful establishment of DM model rats.

### Exercise-induced changes in the body weight and glycolipid metabolism of the obesity and diabetes rats

As shown in Fig. 2, the body weights of OB rats were decreased significantly by 4-week exercise, which were closed to that of the Con rats. When DM rats were established, they had lower body weights than OB rats and similar body weight as Con rats, but decreased progressively after the following 4 weeks, whereas 4-week exercise did not affect the body weights of DM rats (Fig. 2). In addition, the amounts of daily ingestion of these rats were recorded and no significant difference were found between EOB and OB rats (27.7 ± 1.9 g vs 28.1 ± 5.0 g) as well as between EDM and DM rats (60.0 ± 4.3 g vs 67.0 ± 2.7 g), confirmed that the difference between EOB and OB and between EDM and DM rats was exercise alone not involving in diet.

There were disorders of glycolipid metabolism in both OB and DM rats. After 4-week of aerobic exercise, the glycolipid metabolism was improved in the EOB and EDM rats reflected by the attenuations of increased FINS, HOMA-IR, TG and LDL in both EOB and EDM rats, and the improvements of abnormal FBG and TC in EDM rats (Additional file 1, because the effect of exercise on the improvement of glycolipid metabolism was already shown in numerous publications).

### Exercise-induced decreases of serum chemerin and chemerin/CMKLR1 in the liver and gastrocnemius of obesity and diabetes rats

**Fig. 3:**
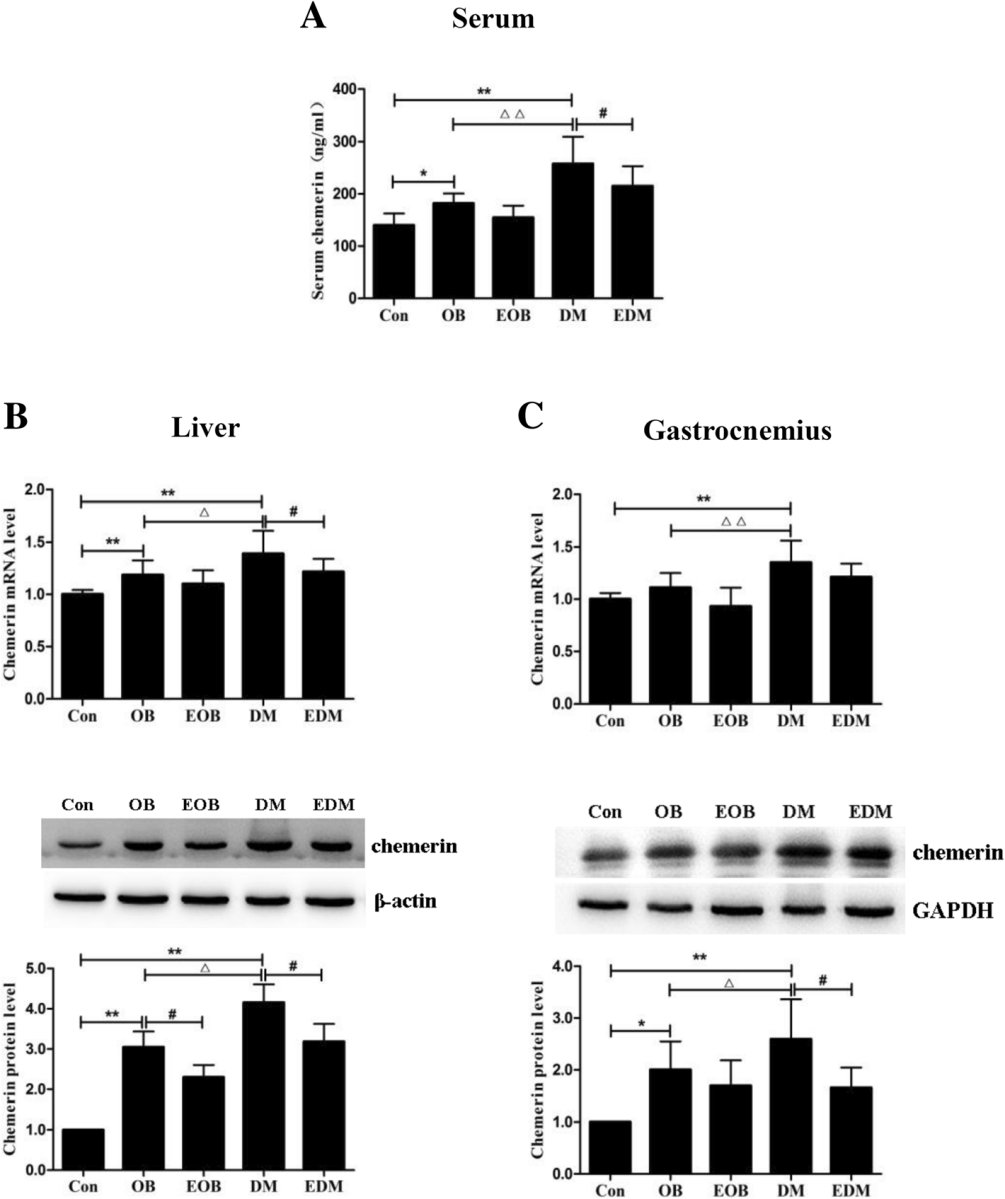
Effects of exercise on the levels of chemerin in serum (**a**), livers (**b**) and gastrocnemius (**c**). Serum levels of chemerin in OB and DM rats were detected by ELISA, and found a decrement of serum chemerin in DM rats rather than OB rats by exercise (**a**). The mRNA and protein levels of chemerin were measured by quantitative real-time PCR and Western blot, respectively. The chemerin in the livers (**b**) and gastrocnemius (**c**) of OB and DM rats were decreased at mRNA (upper) and protein (middle) levels through exercise. The blots of chemerin were quantified by Tanon software and normalized against β-actin or GAPDH, then the normalized numbers were compared between different groups (bottom). OB: obesity; EOB: exercised OB; DM: diabetes mellitus; EDM: exercised DM. ^*^P<0.05, ^**^P<0.01 vs Con. ^#^*P<*0.05, ^##^*P<*0.01 EOB vs OB or EDM vs DM; ^△^*P<*0.05, ^△△^*P<*0.01 DM vs OB

Not only serum chemerin but also the levels of chemerin in peripheral metabolic organs including liver and gastrocnemius (not detecting adipose for no available perirenal adipose from DM rats) were increased significantly in OB and DM rats, with more remarkable increase in DM rats. After 4-week aerobic exercise, the serum chemerin was decreased significantly in EDM rats compared to DM rats, while no obvious reduction in EOB rats compared to OB rats (only a trend, *p* > 0.05). For chemerin in the metabolic organs, 4-week exercise lowered the protein levels of chemerin in both the livers of EOB and EDM rats and the gastrocnemius of EDM rats, while decreased the mRNA levels only in the livers of EDM rats (Fig. 3).

**Fig. 4:**
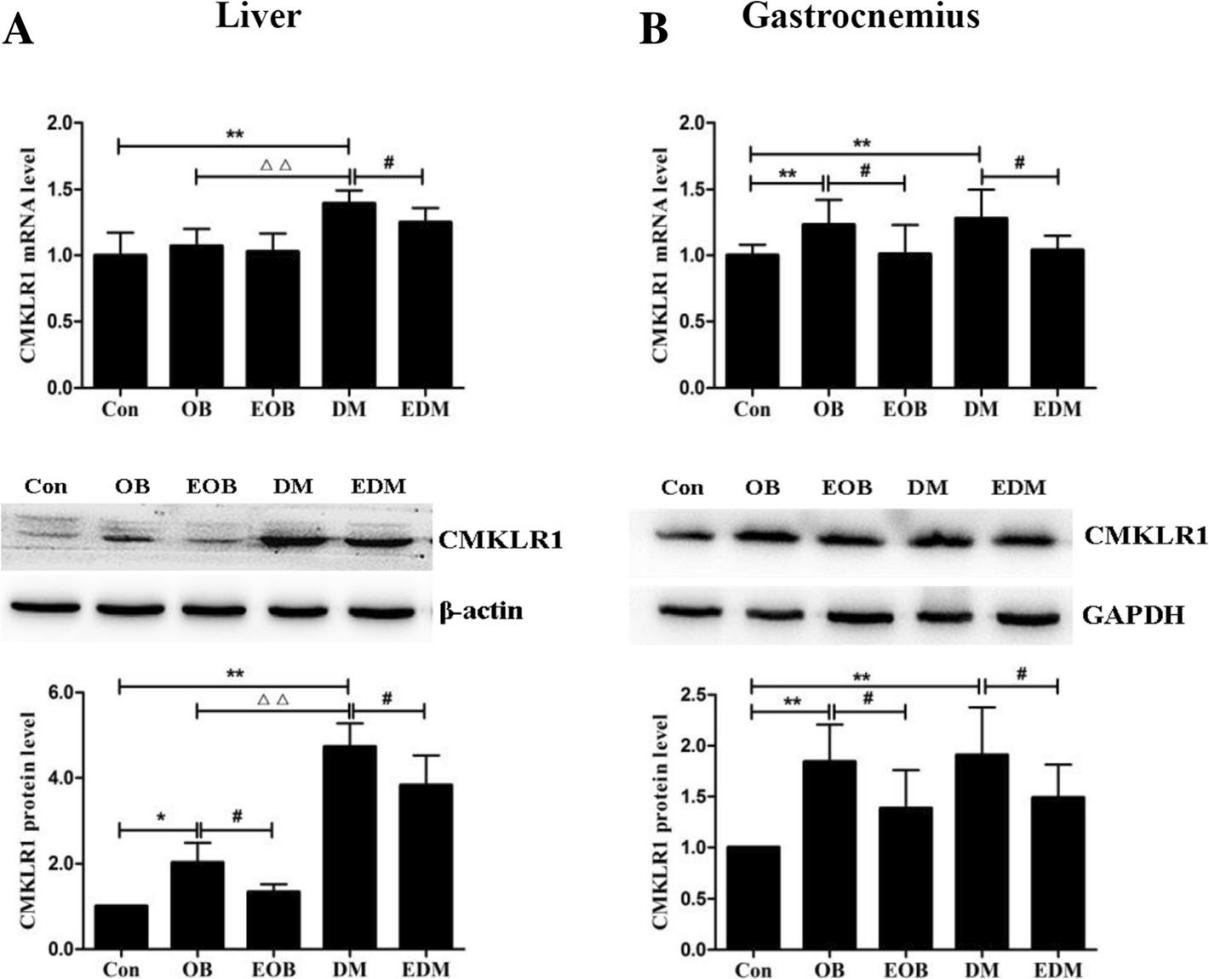
Effects of exercise on the level of CMKLR1 in livers (**a**) and gastrocnemius (**b**). The CMKLR1 in the livers (**a**) and gastrocnemius (**b**) of OB and DM rats was reduced at mRNA (upper) and protein (middle) levels through exercise. The blots of CMKLR1 were quantified by Tanon software and normalized against β-actin or GAPDH, then the normalized numbers were compared between different groups (bottom). OB: obesity; EOB: exercised OB; DM: diabetes mellitus; EDM: exercised DM. ^*^P<0.05, ^**^P<0.01 vs Con. ^#^*P<*0.05, ^##^*P<*0.01 EOB vs OB or EDM vs DM; ^△^*P<*0.05, ^△△^*P<*0.01 DM vs OB

CMKLR1 was also increased in the liver and gastrocnemius of OB and DM rats with higher levels in DM rats, and 4-week aerobic exercise reduced significantly the mRNA and protein levels of CMKLR1 in the liver and gastrocnemius of EOB and EDM rats (Fig. 4).

### Exercise-induced enhancement of PPARγ in the liver and gastrocnemius of obesity and diabetes rats

**Fig. 5:**
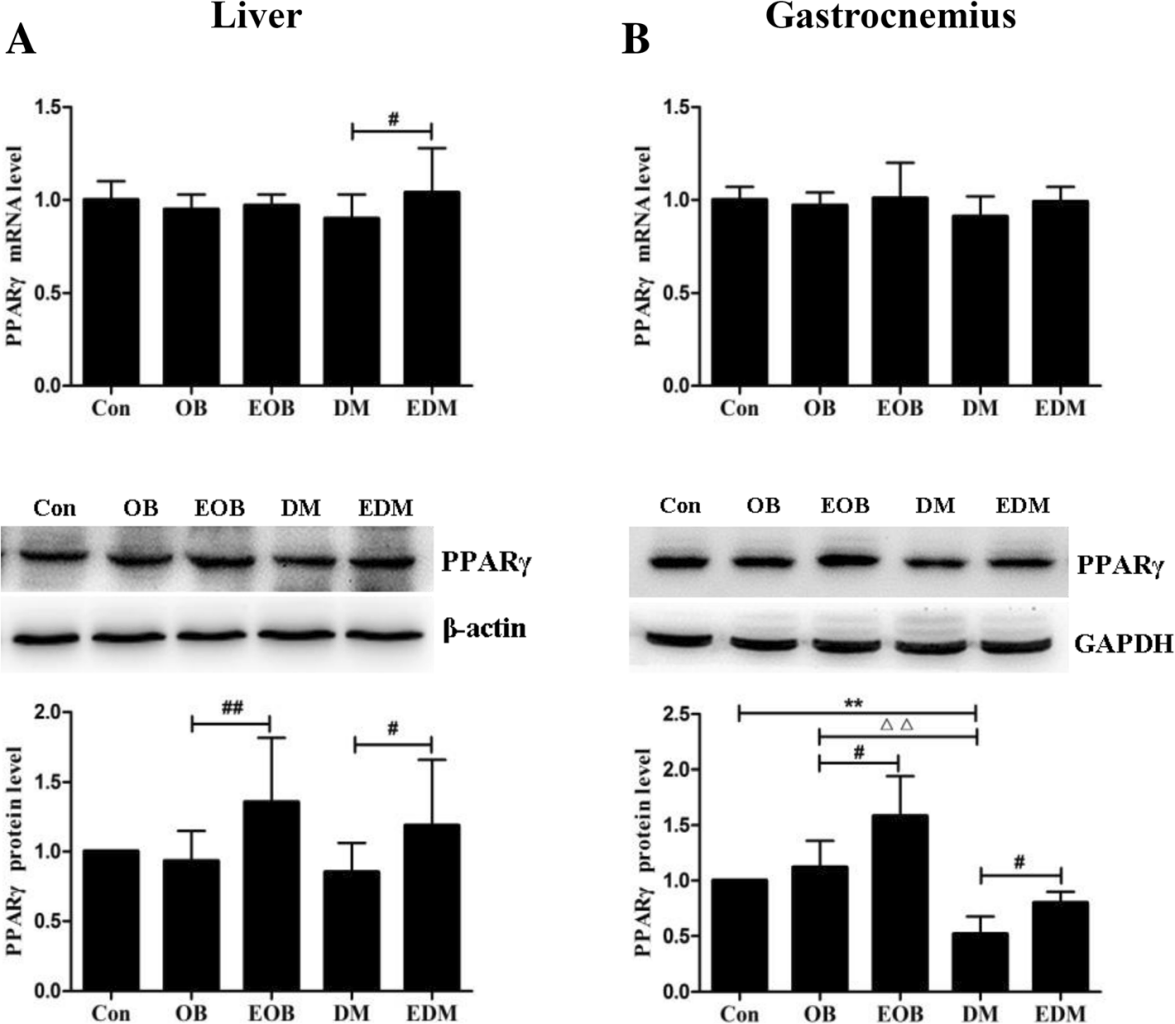
Effects of exercise on the level of PPARγ in livers (**a**) and gastrocnemius (**b**). Exercise promoted PPARγ expression in the livers (**a**) and gastrocnemius (**b**) of OB and DM rats at mRNA (upper) and protein (middle) levels. The blots of PPARγ were quantified by Tanon software and normalized against β-actin or GAPDH, then the normalized numbers were compared between different groups (bottom). OB: obesity; EOB: exercised OB; DM: diabetes mellitus; EDM: exercised DM; ^*^P<0.05, ^**^P<0.01 vs Con. ^#^*P<*0.05, ^##^*P<*0.01 EOB vs OB or EDM vs DM; ^△^*P<*0.05, ^△△^*P<*0.01 DM vs OB

As shown in Fig. 5, 4-week of aerobic exercise enhanced significantly the protein levels of PPARγ in the lives and gastrocnemius of EOB and EDM rats, while increased the mRNA levels only in the livers of EDM rats.

### Exercise-induced attenuations of chemerin/CMKLR1 via the mediation of PPARγ in diabetes rats

**Fig. 6:**
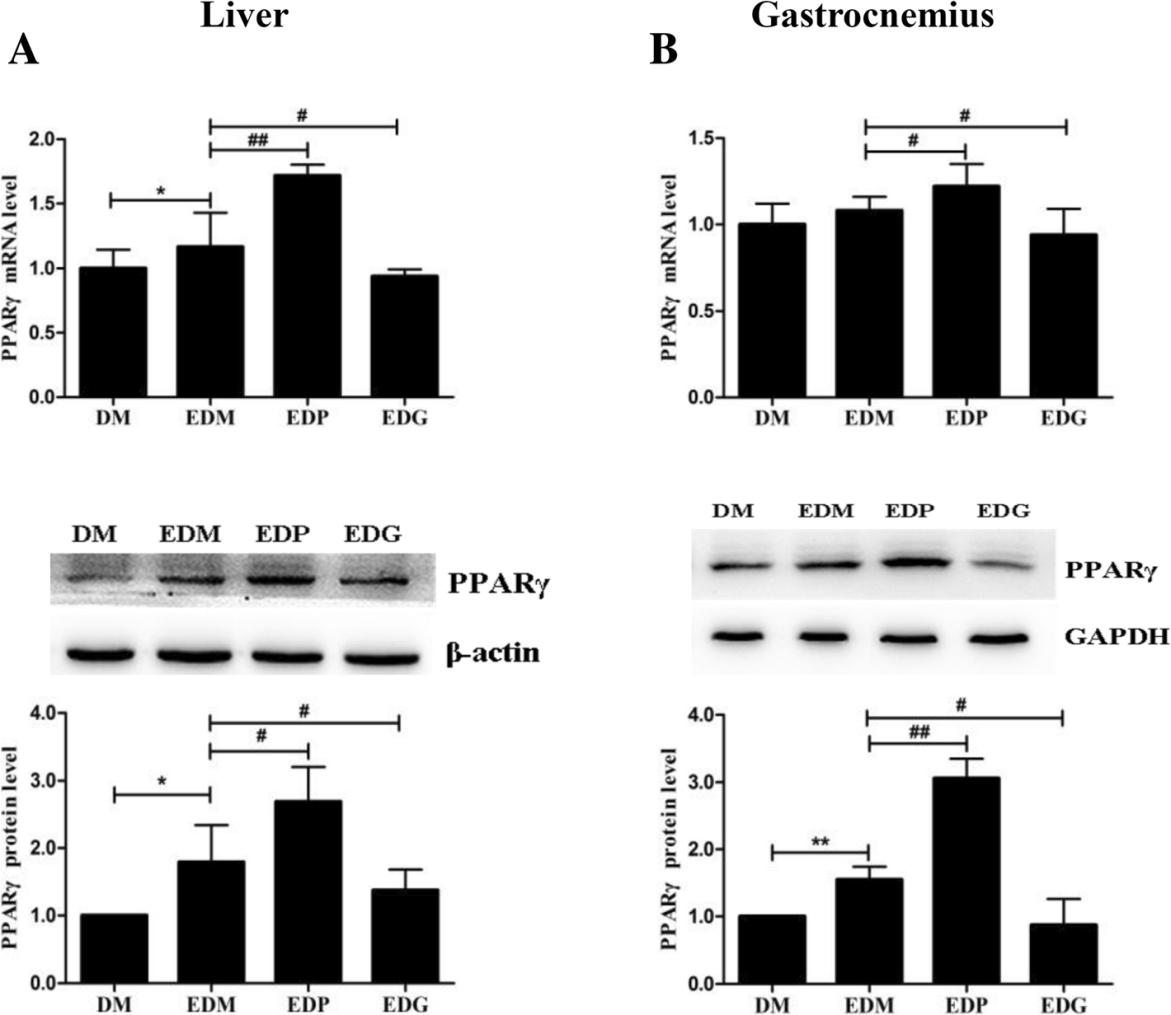
Confirmation of PPARγ agonists pioglitazone and antagonist GW9662 on PPARγ expression in the livers (**a**) and gastrocnemius (**b**) of EDM rats. Exercise-induced increases of PPARγ in the livers (**a**) and gastrocnemius (**b**) of EDM rats were reversed by PPARγ antagonist GW9662 and further increased by PPARγ agonist pioglitazone at mRNA (upper) and protein (middle) levels. The blots of PPARγ were quantified by Tanon software and normalized against β-actin or GAPDH, then the normalized numbers were compared (bottom) between different groups. DM: diabetes mellitus; EDM: exercised DM; EDP: EDM + pioglitazone; EDG: EDM + GW9662. ^*^P<0.05, ^**^P<0.01 vs DM. ^#^*P<*0.05, ^##^*P<*0.01 vs EDM

Pioglitazone and GW9662, the widely used PPARγ agonist and PPARγ antagonist, were demonstrated to promote and inhibit PPARγ signaling in sedentary diabetes rats, respectively. However, if pioglitazone and GW9662 still influenced PPARγ expression in exercised diabetes rats, whose PPARγ expression were increased, have not been confirmed. So at first, the levels of PPARγ were detected after using the common dosages of pioglitazone (10 mg/kg body weight) and GW9662 (1 mg/kg body weight) in the EDM rats, and similar results were found (decrease of PPARγ by GW9662 and further increase of PPARγ by pioglitazone in the livers and gastrocnemius of EDM rats), which indicated the effective regulation of pioglitazone and GW9662 on PPARγ levels in the EDM rats (Fig. 6).

**Fig. 7:**
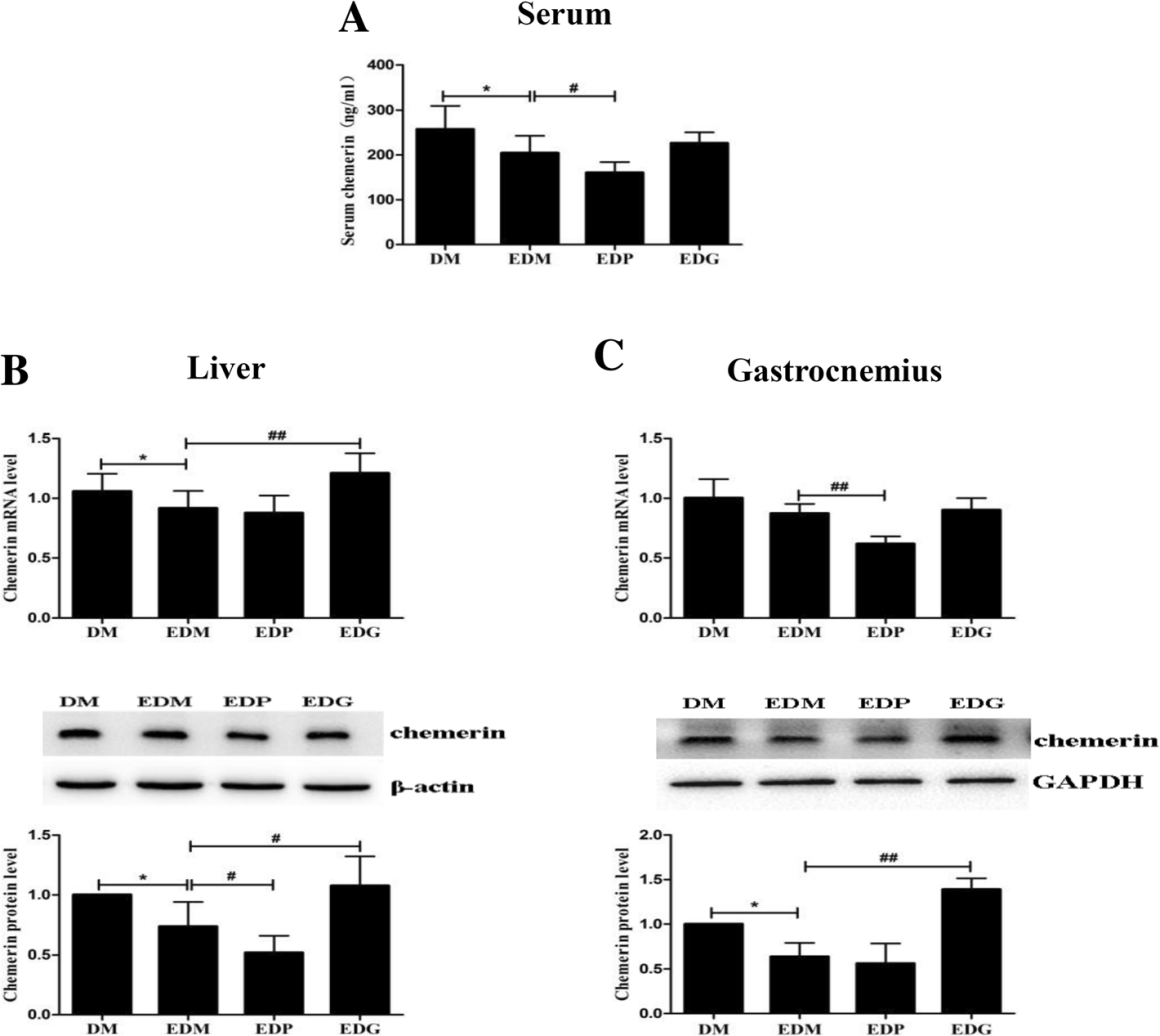
Effects of PPARγ agonist and antagonist on chemerin level in the serums (**a**), livers (**b**) and gastrocnemius (**c**) of EDM rats. PPARγ antagonist GW9662 had no influence on exercise-induced decrease of chemerin in serum (**a**) but significant reversed the decreases of chemerin in the livers (**b**) and gastrocnemius (**c**) of EDM rats at mRNA (upper) and protein (middle) levels. Pioglitazone further strengthened the down-regulations of chemerin in the serums (**a**), livers (**b**) and gastrocnemius (**c**) of EDM rats. The blots of chemerin were quantified by Tanon software and normalized against β-actin or GAPDH, then the normalized numbers were compared between different groups (bottom). DM: diabetes mellitus; EDM: exercised DM; EDP: EDM + pioglitazone; EDG:EDM + GW9662. ^*^P<0.05, ^**^P<0.01 vs DM. ^#^*P<*0.05, ^##^*P<*0.01 vs EDM

**Fig. 8:**
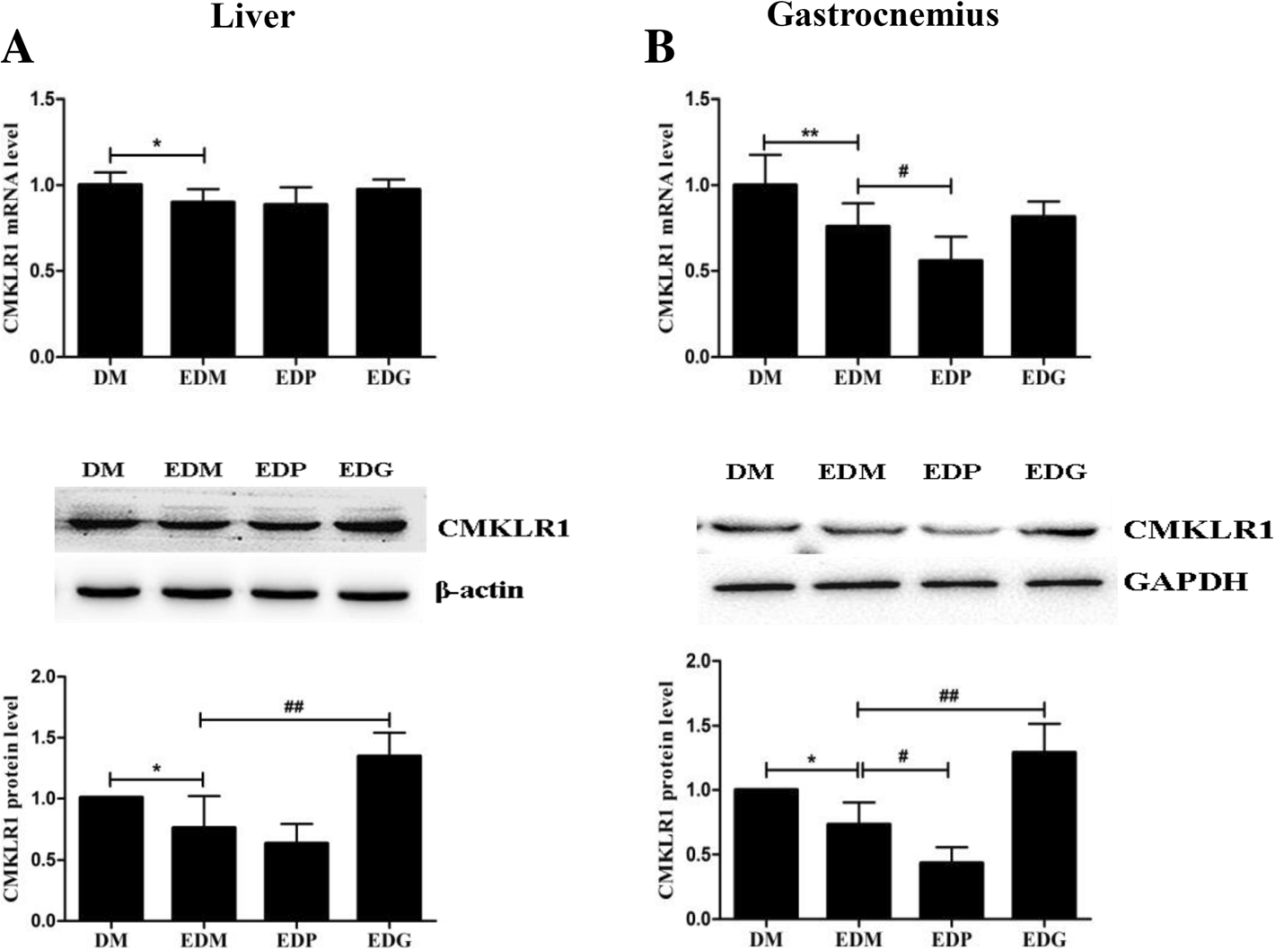
Effects of PPARγ agonist and antagonist on CMKLR1 level in the livers (**a**) and gastrocnemius (**b**) of EDM rats. The exercise-induced decreases of CMKLR1 in the livers (**a**) and gastrocnemius (**b**) of EDM rats were reversed by PPARγ antagonist GW9662 at protein levels. PPARγ agonist pioglitazone further strengthened the reduction of CMKLR1 in the gastrocnemius (**b**) instead of the livers (**a**) of EDM rats. The blots of CMKLR1 were quantified by Tanon software and normalized against β-actin or GAPDH, then the normalized numbers were compared between different groups (bottom). DM: diabetes mellitus; EDM: exercised DM; EDP: EDM + pioglitazone; EDG: EDM + GW9662. ^*^P<0.05, ^**^P<0.01 vs DM. ^#^*P<*0.05, ^##^*P<*0.01 vs EDM

For clarifying the relationship between chemerin/CMKLR1 and PPARγ in the EDM rats, the levels of chemerin and CMKLR1 were detected after treating the EDM rats with pioglitazone or GW9662. As shown in Fig. 7, the exercise-induced decreases of chemerin in the livers and gastrocnemius rather than serum chemerin (only a trend, *p* > 0.05) were reversed by GW9662 in the EDM rats, and pioglitazone further strengthen the attenuations of chemerin in the serum, livers (at protein level) and gastrocnemius (at mRNA level) of the EDM rats. Similarly, the exercise-induced attenuations of CMKLR1 in the livers and gastrocnemius of EDM rats were reversed by GW9662 at protein levels, and the reduction of CMKLR1 in the gastrocnemius not the livers of EDM rats were further strengthen by pioglitazone (Fig. 8).

## Discussion

### Increases of serum chemerin and chemerin/CMKLR1 in peripheral metabolic organs in the obesity and diabetes rats and their associations with glycolipid metabolism

The important roles of enhanced serum chemerin in the development of type 2 diabetes and the disorder of glucose metabolism had been demonstrated thoroughly in the patients with obesity and diabetes [34]. Not only glucose metabolism, serum chemerin also showed positive correlations with potent health threatening components of lipid profile including TC and cholesterol levels in adolescents [35], and promoted cholesterol uptake in human monocyte-derived macrophages which play a role in the development of atherosclerosis and foam cell formation [36]. Besides serum chemerin, the increase of chemerin in the livers of ob/ob mice (obesity model) and db/db mice (diabetes model) was demonstrated a key role in glucose metabolism, and administration of exogenous chemerin exacerbated glucose intolerance and decreased tissue glucose uptake in obese/diabetic mice [37]. The present study broadened the increases of chemerin in the obesity and diabetes rats from serum, liver to gastrocnemius, and found a potential concentration-dependent relationship between chemerin and the disorder of glycolipid metabolism according to the gradual increment of chemerin levels (Con rats<OB rats<DM rats) in the serum and metabolic organs, accompanied with the rising disorders of glycolipid metabolism.

As a adipokine, chemerin has the dual functions of regulating inflammation and glycolipid metabolism through the receptor CMKLR1. Chemerin can recruit dendritic cells and macrophages which abundantly expressing CMKLR1 to the sites of inflammation to play roles in immunity and inflammation. CMKLR1 is also expressed in both adipocytes and stromal vascular cells of adipose tissue, but most highly in mature adiposities, and the increased CMKLR1 in adipose promotes the secretion of chemerin by autocrine, aggregating the infiltration of macrophage in adipose tissue and obesity associated inflammation. The vital roles of chemerin/CMKLR1 on disorders of glycolipid metabolism including IR and obesity associated inflammation were demonstrated by knockout mice deficiency in chemerin or CMKLR1 [14, 38]. The present study confirmed the enhancement of CMKLR1 in the livers and gastrocnemius of obesity and diabetes rats, suggested the possibility of CMKLR1 on the disorder of glycolipid metabolism by increasing inflammation in the lives and muscles.

It’s worth mentioning that the FINS levels in our HFD-STZ induced diabetes rats were not lowered compared to the obesity rats (both 2-fold increase) till the end of the 4-week experiments. It was an unexpected result because of the known impairment role of STZ on insulin β cells. Similar results were reported by other researchers, who reported that the FINS in HFD-STZ induced diabetes rats was still increased by about 2-fold at 4 weeks after 35 mg/kg of STZ injection [39] and at 12 weeks after 45 mg/kg of STZ injection [40]. Elaidy et al. reported that the FINS in HFD-STZ (50 mg/kg) induced diabetes rats was time-dependent, reflected by higher level at the third week after STZ injection while reductions from the forth week post-injection till the end of the study [41]. So we speculated that the high level of FINS in our diabetes rats was likely to be attributed to lower dosage of STZ (30 mg/kg body weight) and short time after STZ injection (4 weeks).

Although most reports including ours supported the promotion effects of chemerin/CMKLR1 on the disorder of glycolipid metabolism and inflammatory, but some studies suggested an anti-inflammatory effect of chemerin/CMKLR1 [42] and a positive effect of CMKLR1 on glycolipid metabolism because CMKLR1(−/−) mice exhibited HFD-induced exacerbation of glucose intolerance, increase of insulin level and promotion of IR [43]. Wargent ET et al. even suggested that CMKLR1 agonist may be better than CMKLR1 antagonist in the treatment of type 2 diabetes [44]. Further studies are needed to clarify the potential dichotomous actions of CMKLR1.

### Decreases of chemerin/CMKLR1 through exercise in obesity and diabetes rats and their associations with the improvement of glycolipid metabolism

It had been demonstrated that serum chemerin was reduced by exercise, such as 12 week Nordic walking in overweight and obese men [21], 6-month combined endurance and strength training in obese adults [23], 1 year regular moderate walking in the patients with type 2 diabetes [22], even an acute bout of aerobic exercise in obese adults [45]. The present study found that as short as 4-week aerobic exercise not only mitigated the serum chemerin of diabetes rats but also decreased the chemerin in the livers of obesity and diabetes rats and in the gastrocnemius of diabetes rats. In addition, the present study first reported, to our knowledge, that 4-week aerobic exercise attenuated the levels of CMKLR1 in the livers and gastrocnemius of obesity and diabetes rats.

Numerous studies have reported beneficial effects of exercise training and/or weight loss on chemerin levels. In the present study, it is unclear whether the decreases of chemerin/CMKLR1 in EOB rats were due to exercise alone or exercise-related weight loss. But for the EDM rats in the study, their body weight did not change significantly and the decreases of chemerin/CMKLR1 were also found compared to the DM rats, suggested a direct effect of exercise (not requiring the mediation of weight loss) on attenuating the chemerin/CMKLR1 in DM rats. Besides, exercise-induced attenuation of chemerin in serum has been demonstrated to alleviate IR and subclinical inflammation in overweight and obese type 2 diabetes patients [46–48], and the present study first broaden the effects of reduced chemerin on the improvement of glycolipid metabolism from serum to peripheral metabolic organs, and first reported the exercise-induced decrease of CMKLR1 in metabolic organs, which was one significance of the present study.

### Exercise-induced reductions of chemerin/CMKLR1 were mediated by PPARγ

As mentioned in introduction, PPARγ has been identified as a therapeutic target for obesity, hyperlipidaemia and diabetes for the dual functions of regulating glycolipid metabolism [28, 29] and inhibiting inflammation [30, 31]. Exercise increased the levels of PPARγ in the livers, skeletal muscle and circulating monocytes, which might be related to the decreases of IR and hepatic lipid content [49] and enhancements of glucose uptake and fatty acid oxidation of skeletal muscle in obese Zucker rats [32], as well as the decrease of IR and prevention of type 2 diabetes in human [31].

Chemerin is a target gene of PPARγ in promoting mesenchymal stem cell adipogenesis [33], and in vitro PPARγ agonist suppressed the release of chemerin from adipocytes and adipose tissue [50] and decreased chemerin expression in adipocytes [33, 51]. In vivo, PPARγ agonist induced reductions of chemerin and/or CMKLR1 in diabetic rats which might be associated with the ameliorations of diabetic nephropathy [52]. Whether there may exist some relation between PPARγ and chemerin/CMKLR1 on regulating glycolipid metabolism in vivo has not been reported. For clarify the question, widely used PPARγ agonist pioglitazone and PPARγ antagonist GW9662 [53] were administrated in the EDM rats at the dosage of 10 mg/kg body weight and 1 mg/kg body weight respectively, not only on the basis of lots of relevant documents [54, 55] but also by our experiment to demonstrate the effectiveness of pioglitazone and GW9662 at the above dosages in regulating PPARγ expression in EDM rats. Then Our study found that exercise-induced decreases of chemerin/CMKLR1 in the livers and gastrocnemius were mediated via PPARγ, and exercise plus PPARγ agonist was more effective than exercise alone in decreasing chemerin/CMKLR1 and improving glycolipid metabolism of diabetes rats. In addition, there was a discrepancy between mRNA level and protein level in the CMKLR1 of EDG rats (protein level increased while mRNA unchanged), and we speculated that the reason for the discrepancy might be involved in the decline of protein degradation. To our knowledge, it is also the first report regarding to PPARγ in mediating exercise-induced attenuations of chemerin/CMKLR1, which is the other significance of the present study.

There are some strengths of our study: (1) broadened the inhibition of chemerin by exercise from circulation to peripheral metabolic organs in diabetes rats and first reported the exercise-induced attenuation of CMKLR1 in metabolic organs. (2) first reported that exercise- induced decreases of chemerin/CMKLR1 in circulation and organs were mediated by PPARγ in diabetes rats. Some limitations need to be considered, for example, the present study does not strongly confirm the roles of chemerin/CMKLR1 on exercise-induced improvement of glycolipid metabolism and its possible mechanism in obesity and diabetes rats, for lacking rat-specific commercial exogenous chemerin as well as agonist and antagonist of CMKLR1.

## Conclusions

**Fig. 9:**
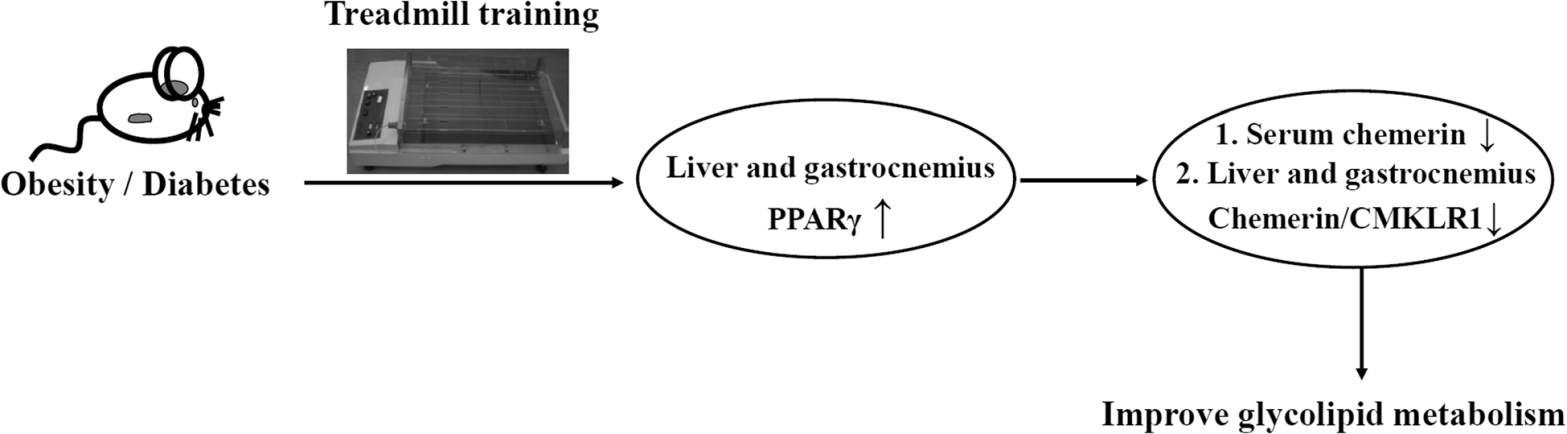
Summary diagram. Besides serum chemerin, 4-week exercise training reduced the levels of chemerin and CMKLR1 in the livers and gastrocnemius of obesity and diabetes rats, which were likely to be associated with the improvement of glycolipid metabolism. The exercise-induced decrements of chemerin/CMKLR1 were mediated by enhancing PPARγ in the livers and gastrocnemius of the diabetes rats

Besides serum chemerin, the levels of chemerin/CMKLR1 in the metabolic organs of obesity and diabetes rats were alleviated by 4-week aerobic exercise, which were likely to be associated with the improvement of glycolipid metabolism. Exercise-induced decrements of chemerin/CMKLR1 in the diabetes rats were mediated by PPARγ (Fig. 9).

## Additional file

Additional file 1: **Figure S1.** Improvement of glucose and lipid metabolism through exercise in obesity and DM rats. The glycolipid metabolism index before and after 4-week exercise intervention were detected including fasting blood glucose (FBG), fasting insulin (FINS), triglyceride (TG), total cholesterol (TC), LDL and HDL. Homeostasis model assessment of insulin resistance (HOMA-IR) was calculated by FBG (mmol/L) × FINS (μU/mL) / 22.5. Con: control; OB: obesity; EOB: exercised OB; DM: diabetes mellitus; EDM: exercised DM. ^*^P<0.05; ^**^P<0.01 vs Con; ^#^*P<*0.01, ^##^*P<*0.01 EOB vs OB or EDM vs DM. (TIF 7212 kb)

## Abbreviations

CMKLR1: Chemerin receptor chemokine-like receptor
FBG: Fasting blood glucose
FINS: Fasting insulin
HDL: High-density lipoprotein cholesterol
HFD: High fat diet
HOMA-IR: Homeostasis model assessment of insulin resistance
LDL: Low-density lipoprotein cholesterol
PPARγ: Peroxisome proliferator activated receptor γ
STZ: Streptozotocin
TC: Total cholesterol
TG: Triglyceride
