# Supplementary figures and images for: Aerobic exercise decreases chemerin/CMKLR1 in the serum and peripheral metabolic organs of obesity and diabetes rats by increasing PPARγ
Source: Nutr Metab (Lond). 2019 Mar 5;16:17. doi: 10.1186/s12986-019-0344-9 (PMC6402136; doi:10.1186/s12986-019-0344-9)

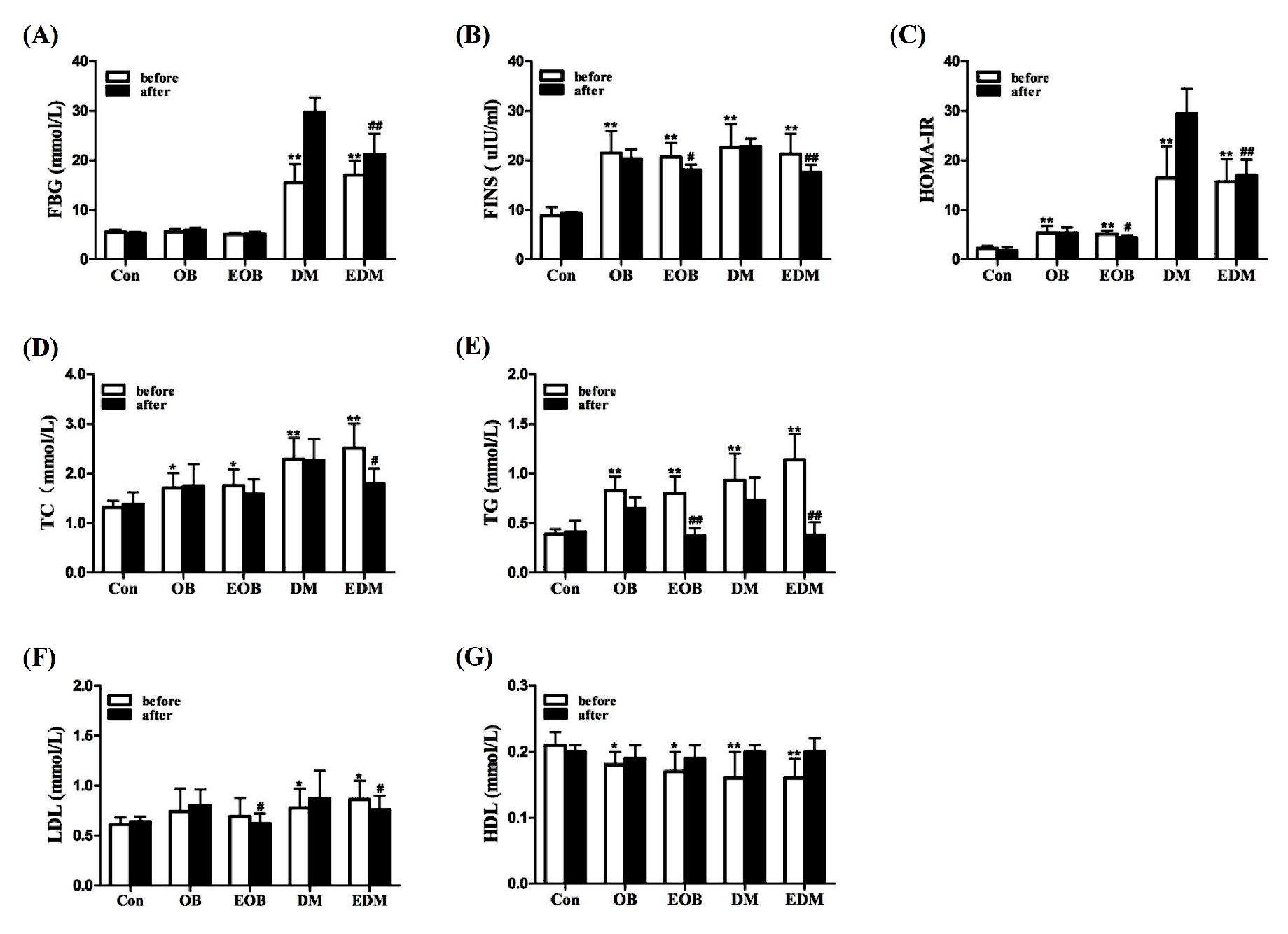

Supplement: Supplementary file 1 — Figure S1. Improvement of glucose and lipid metabolism through exercise in obesity and DM rats. The glycolipid metabolism index before and after 4-week exercise intervention were detected including fasting blood glucose (FBG), fasting insulin (FINS), triglyceride (TG), total cholesterol (TC), LDL and HDL. Homeostasis model assessment of insulin resistance (HOMA-IR) was calculated by FBG (mmol/L) × FINS (μU/mL) / 22.5. Con: control; OB: obesity; EOB: exercised OB; DM: diabetes mellitus; EDM: exercised DM. *P<0.05; **P<0.01 vs Con; #P<0.01, ##P<0.01 EOB vs OB or EDM vs DM. (TIF 7212 kb) [file 12986_2019_344_MOESM1_ESM.tif]
